# Two-stage ligament reconstruction with remnant preservation as treatment of knee dislocation

**DOI:** 10.1186/s12893-023-02271-5

**Published:** 2023-12-08

**Authors:** Wenpu Ma, Yiqun Yang, Xin Ha

**Affiliations:** 1 Department of Orthopaedics, Liaocheng People’s Hospital, No. 67, Dongchang west Road, Liaocheng City, 252000 Shandong Province China; 2 Department of Electromyogram, Liaocheng People’s Hospital, No. 67, Dongchang west Road, Liaocheng City, 252000 Shandong Province China

**Keywords:** Knee dislocation, Anterior cruciate ligament, Posterior cruciate ligament, Ligament reconstruction, Remnant preservation, Arthroscopy

## Abstract

**Objective:**

The purpose of this study was to evaluate the clinical outcomes of two-stage reconstruction (peripheral reconstruction in phase I and central anterior cruciate ligament (ACL) / posterior cruciate ligament (PCL) reconstruction in phase II) with remnant preservation for patients with knee dislocation.

**Methods:**

A total of 70 patients (10 IIIM, 17 IIIL, and 43 IV) with knee dislocation were randomly divided into the remnant-preserved group and the simple reconstruction group. Patients underwent two-stage reconstruction, including the reconstruction of collateral ligament in phase I and the reconstruction of ACL/PCL in phase II (12 weeks after phase I). Grafts were harvested from the semitendinosus and gracilis tendons from both lower limbs. After the surgery, the joint flexion and extension, bone tunnel and ligament healing, and joint stability were evaluated.

**Results:**

After the surgery, the lateral stability recovered in all patients, and X-ray revealed a good position of bone tunnel. Follow-up was performed at 12 months postoperatively and ranged from 24 to 91 months. At the final follow-up, knee flexion angle, IKDC, Lysholm, and Tegner scores were all higher in both groups compared to the preoperative period. Notably, the remnant-preserved group showed superior results in these parameters compared to the simple reconstruction group. There was statistical significance between the two groups in terms of the Lachman test.

**Conclusion:**

The knee function was well recovered after two-stage ligament reconstruction with remnant preservation.

## Introduction

Knee dislocation is an uncommon and severe injury that frequently induced by high-energy impact injuries, ranging from automobile accidents to contact sports [1]. Because knee dislocation is associated with a variety of vascular, neurological, and multi-ligamentous knee injuries, its clinical management requires great attention [2]. In the absence of timely diagnosis and treatment, knee dislocation may even develop to the limb loss [3]. The combined injuries in the anterior cruciate ligament (ACL) and posterior cruciate ligament (PCL) often occur secondary to knee dislocation [4]. Nowadays, cruciate ligament reconstruction based on arthroscopic surgery is still the standard strategy for the treatment of multi-ligament injury [5]. One-stage or multiple staged ligament reconstruction has achieved some progress in improving the outcomes [2]. However, there is no consensus on the best therapeutic strategy, and some patients may experience long-term functional limitations.

Until now, the treatment principle for cruciate ligament injuries following knee dislocation is still controversial. Some scholars recommend that ligament reconstruction should be performed as early as possible after knee dislocation [6, 7]. Li et al. have revealed that one-stage multi-ligament reconstruction is effective to treat Schenck IV knee dislocation, achieving a good postoperative knee function [6]. Hua et al. have found that one-stage reconstruction of ACL/PCL/posterolateral complex combined with MCL (MCL) repair is effective in restoring knee joint stability and improving joint laxity and joint movement in patients with KD-IV knee dislocation [7]. Some other scholars believe that two-stage ligament reconstruction may also have a satisfactory treatment outcome [8, 9]. Inada et al. have indicated that two-stage bicruciate reconstruction of ACL/PCL improves knee stability and self-assessment during follow-up [8]. Ohkoshi et al. have revealed that two-stage reconstruction of ACL/PCL/MCL results in full knee extension without lateral or medial instability in patients with traumatic knee dislocation [9]. In addition, Khan et al. have compared the efficiency of one-stage and two-stage ligament reconstruction for the treatment of multi-ligament knee injury, and have shown similar functional outcomes [10]. However, the successful reconstruction of the cruciate ligament still depends on an accurate and thorough understanding of the pattern of instability imparted by the injury.

Remnant preservation is a technology to make full use of the remnant to improve the surgical outcomes of ligament reconstruction, especially for ACL reconstruction [11]. Because the ACL remnant contains a well-vascularized synovial sheet and a substantial number of fibroblasts, myofibroblasts, and mechanoreceptors, it can promote synovial coverage, revascularization, and enhance the biomechanical properties of grafts [12]. Many studies have reported that remnant-preserving ACL reconstruction achieves significant postoperative improvements in arthroscopic and clinical outcomes [13–15]. In addition, remnant preservation is also beneficial to the stability restoration, graft healing, and proprioception recovery following PCL reconstruction [16, 17]. Therefore, remnant preservation may be valuable in the repair of ACL/PCL injuries secondary to knee dislocation.

In this study, a two-stage reconstruction was performed in patients with knee dislocation. In the phase I, we performed a simple reconstruction of the collateral ligament. When the knee flexion and extension recovered to preoperative range following phase I reconstruction for at least 12 weeks, ACL/PCL reconstruction was subsequently performed. This study aimed to evaluate the clinical effects of two-stage reconstruction of ACL/PCL with remnant preservation, providing a good choice for the treatment of knee dislocation.

## Materials and methods

### Subjects

A total of 70 patients (10 IIIM, 17 IIIL and 43 IV) with knee dislocation were screened at our hospital from May 2010 to August 2020. All patients were randomly divided into two groups: the remnant-preserved group and the simple reconstruction group (Phase I reconstruction + Phase II reconstruction without remnant preservation). In the remnant-preserved group, there were a total of 48 patients, comprising 36 males and 12 females, with an average age of 33.58 ± 7.90 years. Among them, 31 patients had injuries in their left knees, and 17 had injuries in their right knees. The injuries in this group were caused by traffic accidents in 35 cases, sports-related incidents in 7 cases, and falls in 6 cases. The simple reconstruction group included 22 patients, with 17 males and 5 females, and an average age of 34.05 ± 8.17 years. Among these, 15 cases involved left knee injuries, and 7 cases involved right knee injuries. The injuries in this group resulted from traffic accidents in 16 cases, sports-related incidents in 3 cases, and falls in 3 cases. There was no statistical significance between the two groups in terms of age, male, injured part, injury cause, and follow-up time (P > 0.05; Table 1). The inclusion criteria included: (1) a definite history of knee joint trauma; (2) Rupture of ACL and PCL + MCL injury (> grade II) and/or LCL injury (> grade II) were determined by physical examination; (3) Rupture of ACL and PCL + collateral ligament injury were determined by Magnetic Resonance Imaging (MRI); (4) Rupture of ACL and PCL were determined by arthroscopy. The exclusion criteria included: (1) cases not tolerant of surgery due to poor general condition; (2) cases accompanied by vascular/nerve injury and/or fracture; (3) cartilage injury (> Outerbridge grade III); (4) cases underwent cruciate ligament revisions. Before admission, the knee joint has been self-reset or reset by emergency personnel.

**Table 1:** Comparison of general data between the two groups

	Remnant-preserved group (n = 48)	Simple reconstruction group (n = 22)	χ^2^/*t*	P
Age	33.58 ± 7.90	34.05 ± 8.17	-0.225	0.823
Male	36(75%)	17(77%)	0.042	0.837
Injured part			0.087	0.768
Left knee	31(64.6%)	15(68.2%)		
Right knee	17(35.4%)	7(31.8%)		
Injury cause			0.025	0.988
Accident	35(72.9%)	16(72.7%)		
Sport	7(14.6%)	3(13.6%)		
Tumble	6(12.5%)	3(13.6%)		
Follow-up time	57.5 ± 19.9	66.5 ± 10.9	-1.964	0.054

### Phase I reconstruction

In phase I, the collateral ligament was repaired first following detumescence (within 2 weeks of injury). For the avulsion injury of the MCL, the broken end was sutured and fixed at the femoral end or the tibial side using anchor nails, and the substantial fracture was directly sutured. For avulsion injuries of the LCL, the insertion point was fixed directly, and substantial injury or that combined with posterior lateral corner tear was turn-back repair using ipsilateral biceps femoris tendon. Turn-back repair was a surgical procedure in which a damaged or torn tendon was partially detached from its point of insertion, then folded back on itself and reattached. This technique was commonly employed to reinforce repaired tendons and ligaments, enhancing their strength and stability.

All injured knee joints, including those underwent collateral ligament repair, required immobilization in the fully extended position with braces for 3 weeks before starting rehabilitation exercises. After 2 weeks of exercise, the exercise intensity was gradually increased until reaching the maximum tolerable or preoperative range of flexion and extension. Patients began with contact weight-bearing walking using crutches, followed by partial weight-bearing walking during the first 6 weeks, before transitioning to full weight-bearing walking.

### Phase II arthroscopic reconstruction of ACL and PCL

After phase I reconstruction for at least 12 weeks, phase II arthroscopic reconstruction of anterior and posterior cruciate ligaments was performed.

#### Graft acquisition

Semitendinosus and gracilis tendons of both lower limbs were separated from patients as grafts. Both ends of tendons were folded into four strands for backup after measuring the diameter.

#### Bone tunnel establishment

Femoral tunnel- Arthroscopy was performed routinely and concomitant injuries were treated. The adhesion between the anterior and posterior cruciate ligaments was removed to prevent damage to the ligament remnants. The internal opening of the femoral tunnel of the ACL is located at the end point of the anteromedial fasciculus. After cleaning the residual ligament tissues at the end point, the posterior wall of the lateral femoral condyle was exposed. Subsequently, the knee was bent for 120^o^, and a locator was inserted via an anterior internal incision of the knee. The locator tip was positioned at 1:30 of the left knee, and the guide needle was drilled at half position 10:30 on the right knee. The inner part of the femoral tunnel was then established using a femoral tunnel drill of appropriate diameter. The outer part of the femoral tunnel is established using a 4.5 mm Retrobutton drill.

**Fig. 1:**
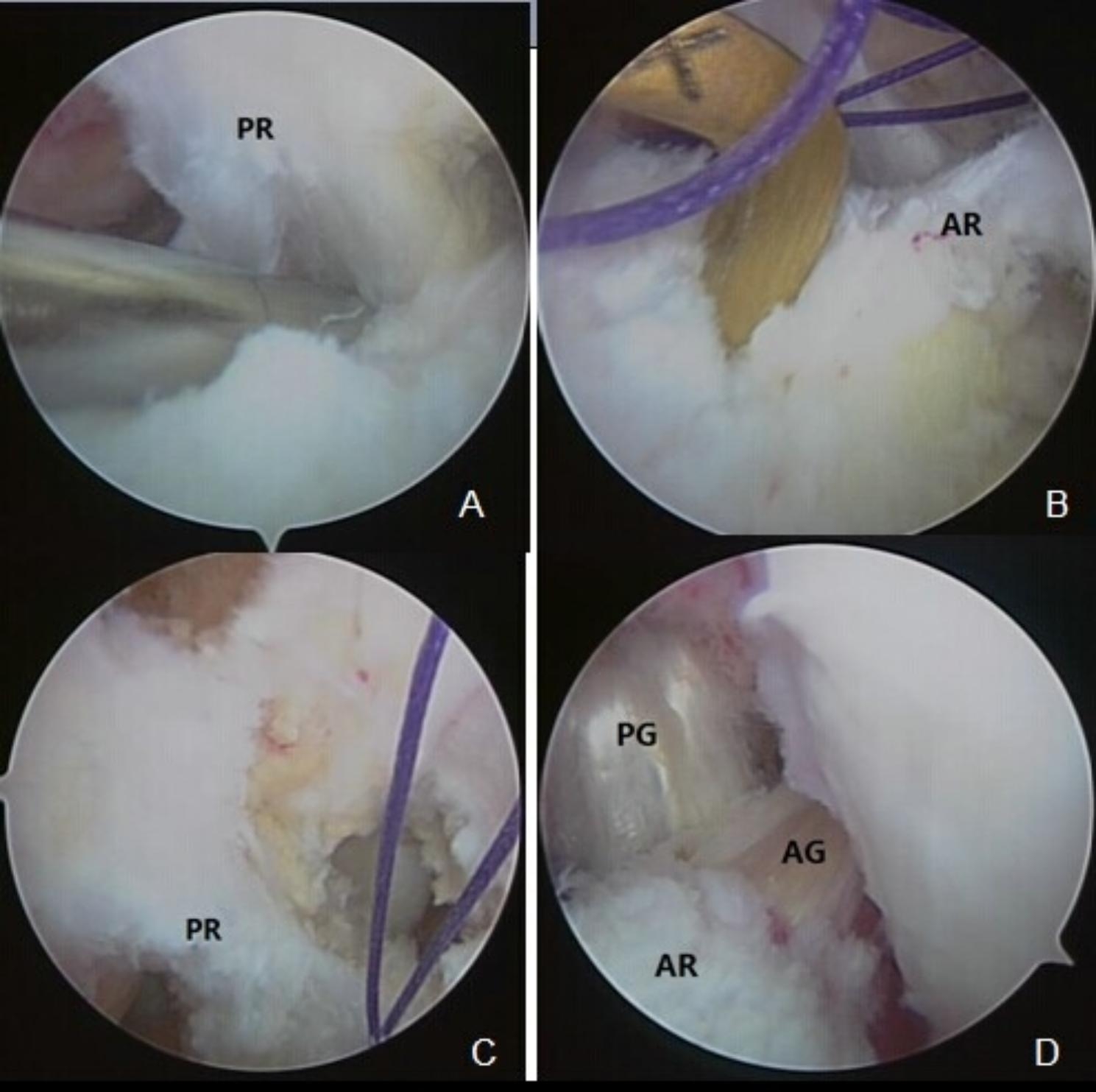
Important steps in the reconstruction of ACL and PCL. **A** The attachment point of PCL tibial remnant (PR) was stripped and pushed to the rear of the knee joint using a stripper. **B** The internal opening of ACL tibial tunnel is located within the ACL remnant (AR) (AR was conserved). **C** The inner opening of the PCL femoral tunnel was located at the anteromedial fasciculus of PR (PR was conserved). **D** The tibial lateral part of ACL graft (AG) was covered by PCL graft (PG) after reconstruction

PCL tibial tunnel- After bending the knee for 90^o^, arc PCL stripper was inserted along an anterior internal incision of the knee, and the ending point of PCL at the anterior, medial, and posterior shin knee as well as surrounding joint capsule were stripped (Fig. 1A). Subsequently, PCL tibial tunnel locator (Smith&Nephew) was placed via anterior medial entrance under arthroscope, at an angle between 50° and 55°. The locator tip was positioned at the ending point of PLC as low as possible. Followed by inserting of Kirschner needle, appropriate depth limiting bit was used to establish the PCL tibial tunnel according to the diameter of graft.

ACL tibial tunnel- ACL tibial tunnel locator (Smith&Nephew) was also placed via anterior medial entrance at an angle between 40° and 45°. The locator tip was positioned in the non-impact area at internal posterior side of original ACL tibia attachment remnant (Fig. 1B). The outer opening of ACL tibial tunnel was on the inner side of the midline, distancing from the outer opening of PCL tunnel for more than 10 mm. Along the inserted guide needle, a bone tunnel was drilled until the tibial remnant. In order to avoid the damage on the ligament remnant, drilling was stopped when penetrating bone cortex.

Outer part of tunnel-The inner opening of the PCL femoral tunnel was located at the anterolateral bundle end of the PCL footprint (Fig. 1C), positioned approximately 7–8 mm away from the distal articular cartilage edge. A guide needle was drilled to guide the hollow drill, and the outer part of tunnel was drilled using a 4.5 mm Retrobutton drill. The lead wire is threaded through the double tunnel using a lead wire needle and a tibial guide wire.

#### Graft fixation

Under arthroscopy, the PCL/ACL-Retrobutton compound was pulled into the joint cavity through the tibial tunnel, and then into the femoral tunnel with a traction line. The Retrobutton was fixed after the femoral end was turned over. When the Drawer and Lachman tests were negative, the knee was bent for 70^o^ to tension the graft, and the external opening of the PCL tunnel was then fixed using interference screws. The external opening of the ACL tunnel was fixed with interface screws at 30^o^ of knee flexion. After fixation, the stability of the knee joint was examined under arthroscopy and physical examination (Fig. 1D). Finally, the incision was closed, and the knee joint was fixed using locking brace in a straight position.

### Postoperative rehabilitation exercise

A mild rehabilitation plan was applied according to the rehabilitation of PCL. To protect the reconstructed PCL during rehabilitation exercises, shinbone tenesmus should be avoided. From the second week post-surgery, knee flexion training was performed twice a day and gradually increased to 90°. The knee flexion reached to 120° within 8 weeks and then expanded to its maximum degree. Only contact weight-bearing exercises with crutches were allowed within 6 weeks after the surgery, and full weight-bearing exercises with adjustable support of mathematical chuck were performed after the surgery for 10 weeks. Swimming and other mild exercises were allowed at 3 months post-surgery, jogging was allowed at 6 months post-surgery, and strenuous exercises were allowed at 12 months post-surgery.

### Postoperative evaluations

After the surgery for 12 months, the symptoms, range and stability of joint flexion and extension, Lysholm score, Tegner score, and IKDC score were evaluated. X-ray and MRI were performed to reveal the changes of bone tunnel and ligament healing. Lachman, Drawer, Valgus/Varus stress, and axial displacement tests were performed to evaluate the stability of the knee joint.

## Results

### Collateral ligament reconstruction in patients

Within 2 weeks after the injury of collateral ligament of knee joint, repair surgery was performed in patients in different ways, including the posterolateral structure of knee joint (N = 12), lateral termination of the fibula (N = 8), ipsilateral partial biceps femoris tendon reentry (N = 15), MCL (suture of broken end and anchor fixation of broken end; N = 45), and MCL + LCL (suture of broken end and anchor fixation of broken end; N = 5). The duration from when the patient received treatment for the injury to the conclusion of phase II reconstruction amounted to 14.28 weeks (12–21 weeks).

### Postoperative outcomes

No intraoperative and postoperative complications were observed in all patients. After the surgery, the lateral stability recovered in all patients. In the remnant-preserved group, the remnants of both the ACL and PCL were retained. The postoperative knee flexion angle was 132.4 ± 5.1^o^, which was slightly higher than that before reconstruction (128.3 ± 6.6^o^). Restricted knee extension was not observed in all cases during follow-up. However, in the remnant-preserved group, one case had local pain in the distal medial femur due to heterotopic ossification in the femoral side. This pain was gradually relieved at 5 months post-surgery without special treatment.

### Postoperative imaging

**Fig. 2:**
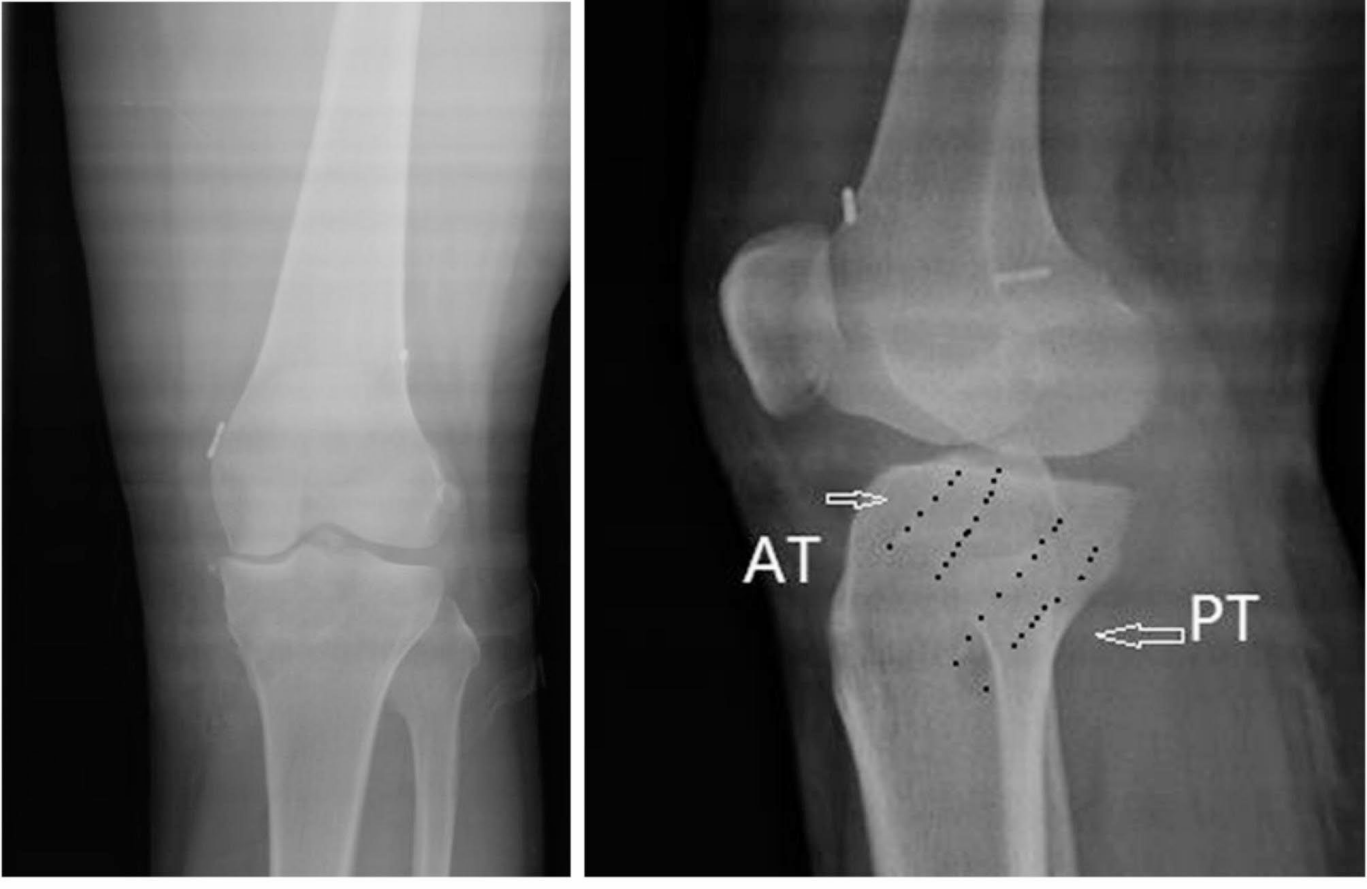
X-ray images revealed that the internal orifice of anterior cruciate ligament tunnel (AT) and posterior cruciate ligament tunnel (PT) on the lateral radiograph is similar to the tibial attachment point of the normal anterior and posterior cruciate ligament after the surgery

**Fig. 3:**
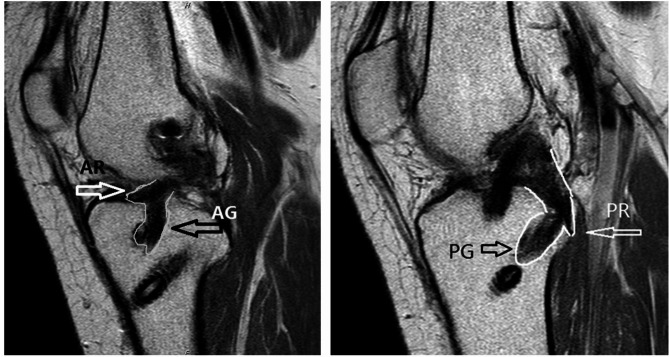
MRI images revealed good fusion of ligamentous remnant with anterior cruciate ligament graft (AG) and posterior cruciate ligament graft (PG) after the surgery

After the surgery, X-ray revealed a good position of the bone tunnel, and the position of the internal orifice of ACL tunnel (AT) and PCL tunnel (PT) is consistent with the normal attachment position (Fig. 2). In addition, postoperative MRI showed good fusion of ligamentous remnant with ACL graft (AG) and PCL graft (PG). The remnant of PCL was pushed to the back of the knee joint following reconstruction (Fig. 3). No cyclops sign was observed in the remnant.

#### Follow-up evaluation

All enrolled 70 cases were followed up for 24–91 months (average 60.3 ± 17.9 months). Follow-up results showed that all patients had no intraoperative and postoperative complications. Compared with the preoperative period, the knee flexion angle, IKDC score, Lysholm score, and Tegner score were significantly improved in both groups at the last follow-up (P < 0.05). In addition, the knee flexion angle, IKDC score, Lysholm score, and Tegner score of patients in the remnant-preserved group were significantly higher than those of patients in the simple reconstruction group at the last follow-up (P < 0.05; Table 2). Before the surgery, the Lachman test and Drawer test were conducted, and they all yielded positive results for all patients in both groups. Regarding the stress test, it showed Valgus stress test positive results in 33 patients and the Varus stress test in 17 patients in the remnant-preserved group. Similarly, there were 15 patients with the positive Valgus stress test and 5 patients with the positive Varus stress test in the simple reconstruction group. At the final follow-up, the outcomes of Lachman’s test indicated 38 negative cases and 10 positive cases in the remnant-preserved group, while the simple reconstruction group showed 15 negative cases and 7 positive cases. The differences between the two groups were statistically significant (P < 0.05). The results of the Drawer test showed that in the remnant-preserved group, there was 15 negative cases, 26 cases with Grade I laxity, and 7 cases with Grade II laxity. In the simple reconstruction group, there was 5 negative cases, 14 cases with Grade I laxity, and 3 cases with Grade II laxity. Between the two groups, there was no statistically significant difference (P > 0.05). The results of the Valgus stress test showed that there was no statistically significant difference between the two groups (P > 0.05). There were 22 negative cases and 11 cases with Grade I laxity in the remnant-preserved group. In the simple reconstruction group, 8 cases were negative, and 7 cases had Grade I laxity. The results of the Varus stress test showed that in the remnant-preserved group, 13 patients showed negative and 4 patients showed Grade I laxity. In the simple reconstruction group, there were 3 negative cases and 2 cases of Grade I laxity. There was no statistical significance between the two groups (P > 0.05; Table 3).

**Table 2:** Comparison of knee joint function scores between the two groups

	Remnant-preserved group (n = 48)	Simple reconstruction group (n = 22)	*t*	P
**Knee flexion angle**				
Preoperative	125.4 ± 6.7	120.8 ± 3.8	0.722	0.473
Last follow-up	131.6 ± 4.9	127.2 ± 3.9	3.674	0.000
*t*	8.186	6.370		
P	0.000	0.000		
**IKDC score**				
Preoperative	28.9 ± 4.5	30.1 ± 4.1	0.333	0.292
Last follow-up	88.9 ± 4.9	84.4 ± 5.6	0.838	0.001
*t*	68.640	39.957		
P	0.000	0.000		
**Lysholm score**				
Preoperative	17.1 ± 4.1	16.7 ± 5.7	0.332	0.741
Last follow-up	91.5 ± 3.9	86.7 ± 5.4	4.218	0.000
*t*	83.779	52.224		
P	0.000	0.000		
**Tegner score**				
Preoperative	4.8 ± 0.9	4.4 ± 0.7	1.892	0.063
Last follow-up	5.6 ± 0.9	4.9 ± 0.5	3.353	0.001
*t*	8.517	3.924		
P	0.000	0.001		

**Table 3:** Comparison of knee stability between the two groups at the last follow-up

	Remnant-preserved group	Simple reconstruction group	χ^2^	P
Lachman test			4.481	0.034
Negative	38(79.2%)	12(54.5%)		
Positive	10(20.8%)	10(45.5%)		
Drawer test			0.630	0.730
Negative	15(31.3%)	5(22.7%)		
I	26(54.2%)	14(63.4%)		
II	7(14.6%)	3(13.6%)		
Valgus stress test			0.782	0.376
Negative	22(66.7%)	8(53.3%)		
I	11(33.3%)	7(46.7%)		
Varus stress test			0.528	0.467
Negative	13(76.5%)	3(60%)		
I	4(23.5%)	2(40%)		

## Discussion

In this study, a total of 70 patients underwent a two-stage reconstruction. Following the two-stage reconstruction surgery, all patients regained lateral stability, and no restricted knee extension was observed. The postoperative knee flexion angle recovered to 132.4 ± 5.1^o^. MRI showed a good fusion of ligamentous remnant with grafts. Follow-up results showed that the knee flexion angle, IKDC score, Lysholm score, and Tegner score were significantly improved in both groups at the last follow-up compared to the preoperative period. In addition, preservation of ACL and PCL remnants further improved these indicators of knee dislocation. The Lachman test revealed statistical significance between the remnant-preserved group and simple reconstruction group. Additionally, the results of Drawer test, Valgus stress test, and Varus stress test showed no statistical difference between the two groups.

A two-stage reconstruction strategy can reduce the operative time and decrease the occurrence of stiff knee [18]. We believe that collateral ligament is important for the stability of the knee joint. The staged strategy can reduce the post-operative pain and the knee joint can get an easier recovery of range of motion. Early repair should be performed for knee dislocation associated with a III degree of MCL injury, because early repair, involving a simple suture of the broken ends, is preferable to later complex reconstruction [19]. In addition, no patients underwent staged reconstruction developed knee joint adhesion, and no manual massage was needed for adhesion release. After the staged surgery, all patients showed a good range of motion without knee joint adhesion, which may be attributed to that the patients had a time of 2–4 months to restore the range of motion rom following collateral ligament reconstruction. In this study, none of the patients experienced intraoperative or postoperative complications after two-stage reconstruction. However, potential complications and adverse events are also associated with two-stage reconstruction. The study has shown that the combination of ACL with open reconstruction of the medial collateral ligament increases the incidence of postoperative loss of mobility in patients, with rates going as high as 13% [20]. In addition, incomplete healing of the collateral ligament may lead to residual valgus laxity, which in turn increases the risk of ACL/PCL graft failure. Two-stage reconstructive treatment prolongs the overall rehabilitation period, and during the healing of Phase I collateral ligament repair, the knee may remain unstable, potentially causing further intra-articular injury [21]. Therefore, these complications and disadvantages require further research to overcome in the future.

Histological findings on ACL reveal that the native mechanoreceptors and neural fibres mainly present within the synovial membrane around tibial insertion of the ACL [22]. ACL may play not only a biomechanical, but also a neurophysiological role in maintaining knee homeostasis [23, 24]. According to previous clinical findings, preservation of remnant fibers on reconstructing ACL/PCL results in better outcomes than cruciate ligament reconstruction alone [25, 26]. Adachi et al. reported that preservation of the remnants of ACL at the time of ACL reconstruction achieves better knee sensory and stability than ACL reconstruction alone [25]. Similarly, Lee et al. indicated that remnant-preserving technique achieves good proprioceptive and functional outcomes during ACL reconstruction [26]. In ACL reconstruction, neither the tendon remnant retraction technique nor the simple remnant retention technique can improve the function and stability of the knee joint [27]. Retention of the remnant may lead to cyclops sign after the surgery [28]. Tendon fibers at the remnant end do not travel normally after tensioning, and the end of the remnant is unable to reattach itself to the original footprint of femoral condylar ligament due to contracture and poor blood supply. Abnormal remnant position can even cause impingement symptoms. Here, we attempted to reconstruct the ligament as much as possible within the remnant sheath, which reduces the possibility of inter-patellar impingement in comparison with the technique of remnant retention combined with distraction. Compared with the reconstruction in phase I, phase II reconstruction can help the recovery and retention of residual ligament bundles to a greater extent, especially for the PCL. During PCL reconstruction at the tibial level, we dissect the PCL along with the joint capsule at the posterior aspect as a unit. This posterior movement increases the distance between the tibial insertion of the PCL and the posterior popliteal nerve vessels, ultimately enhancing the safety of the surgery [29]. The safety of the surgery can be further increased because the remnant of PCL acts as a protective barrier in establishing the tibial tunnel.

Clinically, it is important to regain the correct position of the femur-tibia, but it is difficult to achieve this in the reconstruction of ACL combined with PCL, since the only way to control the relative position of the femur-tibia during surgery is to adjust the tension of the transplanted tendons [30]. In order to better acquire the neutral position of the joint, we simultaneously pulled and tightened the ACL/PCL grafts into the bone tunnels, and the tension of the two grafts was repeatedly adjusted when the Drawer and Lachman tests were both negative. Subsequently, the tibia side is fixed by absorbable compression screws. Our surgical strategy finally achieved satisfactory clinical results.

However, there are some limitations in this study. Firstly, the primary cause of knee dislocation in these cases was traffic accidents, which might indicate that therapeutic strategy used in this study was more applicable to the cases with high velocity trauma. Then, the number of retrospective nature and cases was low. Additionally, this was a single-center study, as other co-research centers had a low number of cases reported. In conclusion, although the treatment on simultaneous injuries of the ACL and PCL is challenging, a good clinical outcome is obtained by two-stage reconstruction of ACL/PCL with remnant preservation. This surgery is conducive to the recovery and preservation of the residual ligament bundles, as well as the postoperative recovery of knee joint function, without increasing the risk of incorrect positioning of the bone tunnel.

## Data Availability

This study did not use public datasets. All data in the manuscript is available through the responsible corresponding author.

## References

[1] Stannard JP, Schreiner AJ (2020). Vascular injuries following knee dislocation. J Knee Surg.

[2] Anazor FC, Baryeh K, Davies NC (2021). Knee joint dislocation: overview and current concepts. Br J Hosp Med (Lond).

[3] Gomez-Bermudez SJ, Vanegas-Isaza D, Herrera-Almanza L, Roldan-Tabares MD, Coronado-Magalhaes G, Fernandez-Lopera JF (2021). Vascular injury associated with knee dislocation. Acta Ortop Mex.

[4] Levy BA, Boyd JL, Stuart MJ (2011). Surgical treatment of acute and chronic anterior and posterior cruciate ligament and lateral side injuries of the knee. Sports Med Arthrosc Rev.

[5] Held M, Laubscher M, von Bormann R, Richter DL, Wascher DC, Schenck RC (2021). Open approaches for cruciate ligament reconstruction in knee dislocations: a technical note and case series. SICOT J.

[6] Li C, Liu Y, Zheng R, Sun J, Peng W, Deng XH et al. One-stage arthroscopic multiple ligament Reconstruction for Schenck IV knee dislocation. Orthop Surg. 2022. 10.1111/os.13611 PMC9891907 36513497

[7] Hua W, Liu S, Wang B (2022). Short-term effectiveness of one-stage anterior and posterior cruciate ligaments and posterolateral complex reconstruction combined with medial collateral ligament repair for KD-IV knee dislocation. Zhongguo Xiu Fu Chong Jian Wai Ke Za Zhi.

[8] Inada MM, Piedade SR (2021). Clinical outcomes after two-stage bicruciate knee Ligament Reconstruction. Acta Ortop Bras.

[9] Ohkoshi Y, Nagasaki S, Shibata N, Yamamoto K, Hashimoto T, Yamane S (2002). Two-stage reconstruction with autografts for knee dislocations. Clin Orthop Relat Res.

[10] Khan MJ, Asif N, Sharma A, Siddiqui YS, Khan AQ (2022). Single-stage versus two-stage reconstruction in chronic multi ligament knee injury. Int J Burns Trauma.

[11] Chambat P, Guier C, Sonnery-Cottet B, Fayard JM, Thaunat M (2013). The evolution of ACL reconstruction over the last fifty years. Int Orthop.

[12] Wang H, Liu Z, Li Y, Peng Y, Xu W, Hu N (2019). Is Remnant Preservation in Anterior Cruciate Ligament Reconstruction Superior to the standard technique? A systematic review and Meta-analysis. Biomed Res Int.

[13] Papalia R, Maffulli N, Denaro V (2013). The anterior cruciate ligament remnant: to leave it or not?. Arthroscopy.

[14] Lu W, Wang D, Zhu W, Li D, Ouyang K, Peng L (2015). Placement of double tunnels in ACL Reconstruction using Bony landmarks versus existing footprint remnant: a prospective clinical study with 2-Year follow-up. Am J Sports Med.

[15] Kondo E, Yasuda K, Onodera J, Kawaguchi Y, Kitamura N (2015). Effects of Remnant tissue preservation on clinical and arthroscopic results after anatomic double-bundle Anterior Cruciate Ligament Reconstruction. Am J Sports Med.

[16] Zhao J (2021). Single-bundle anatomical posterior Cruciate Ligament Reconstruction with Remnant Preservation. Arthrosc Tech.

[17] Eguchi A, Adachi N, Nakamae A, Usman MA, Deie M, Ochi M (2014). Proprioceptive function after isolated single-bundle posterior cruciate ligament reconstruction with remnant preservation for chronic posterior cruciate ligament injuries. Orthop Traumatol Surg Res.

[18] Mook WR, Miller MD, Diduch DR, Hertel J, Boachie-Adjei Y, Hart JM (2009). Multiple-ligament knee injuries: a systematic review of the timing of operative intervention and postoperative rehabilitation. J Bone Joint Surg Am.

[19] Vicenti G, Solarino G, Carrozzo M, De Giorgi S, Moretti L, De Crescenzo A (2019). Major concern in the multiligament-injured knee treatment: a systematic review. Injury.

[20] Magit D, Wolff A, Sutton K, Medvecky MJ (2007). Arthrofibrosis of the knee. J Am Acad Orthop Surg.

[21] Holuba K, Vermeijden H, Yang X, O’Brien R, van der List J, DiFelice GJA et al. Treating combined anterior cruciate ligament and medial collateral ligament injuries operatively in the Acute setting is potentially advantageous. 2023;39(4):1099–107. 10.1016/j.arthro.2022.06.023 35817377

[22] Musahl V, Karlsson J (2019). Anterior cruciate ligament tear. N Engl J Med.

[23] Zimny ML, Schutte M, Dabezies E (1986). Mechanoreceptors in the human anterior cruciate ligament. Anat Rec.

[24] Nyland J, Huffstutler A, Faridi J, Sachdeva S, Nyland M, Caborn D (2020). Cruciate ligament healing and injury prevention in the age of regenerative medicine and technostress: homeostasis revisited. Knee Surg Sports Traumatol Arthrosc.

[25] Adachi N, Ochi M, Uchio Y, Sumen Y (2000). Anterior cruciate ligament augmentation under arthroscopy. A minimum 2-year follow-up in 40 patients. Arch Orthop Trauma Surg.

[26] Lee BI, Kwon SW, Kim JB, Choi HS, Min KD (2008). Comparison of clinical results according to amount of preserved remnant in arthroscopic anterior cruciate ligament reconstruction using quadrupled hamstring graft. Arthroscopy.

[27] Jung YB, Jung HJ, Siti HT, Lee YS, Lee HJ, Lee SH (2011). Comparison of anterior cruciate ligament reconstruction with preservation only versus remnant tensioning technique. Arthroscopy.

[28] Noailles T, Chalopin A, Boissard M, Lopes R, Bouguennec N, Hardy A (2019). Incidence and risk factors for cyclops syndrome after anterior cruciate ligament reconstruction: a systematic literature review. Orthop Traumatol Surg Res.

[29] Ahn JH, Wang JH, Lee SH, Yoo JC, Jeon WJ (2007). Increasing the distance between the posterior cruciate ligament and the popliteal neurovascular bundle by a limited posterior capsular release during arthroscopic transtibial posterior cruciate ligament reconstruction: a cadaveric angiographic study. Am J Sports Med.

[30] Markolf KL, O’Neill G, Jackson SR, McAllister DR (2003). Reconstruction of knees with combined cruciate deficiencies: a biomechanical study. J Bone Joint Surg Am.

